# Prognostic Value of the Red Cell Distribution Width-to-eGFR Ratio (RGR) Across Chronic Heart Failure Phenotypes: A Retrospective Observational Pilot Study

**DOI:** 10.3390/jcm14082852

**Published:** 2025-04-21

**Authors:** Andreea Varga, Liviu Cristescu, Marius-Stefan Marusteri, Razvan Gheorghita Mares, Dragos-Gabriel Iancu, Radu Adrian Suteu, Raluca-Maria Tilinca, Ioan Tilea

**Affiliations:** 1 Faculty of Medicine in English, George Emil Palade University of Medicine, Pharmacy, Science and Technology of Targu Mures, 540142 Targu Mures, Romania; andreea.varga@umfst.ro; 2 Doctoral School, George Emil Palade University of Medicine, Pharmacy, Science and Technology of Targu Mures, 540142 Targu Mures, Romania; dragos-gabriel.iancu@umfst.ro; 3 Faculty of Medicine, George Emil Palade University of Medicine, Pharmacy, Science and Technology of Targu Mures, 540142 Targu Mures, Romania; marius.marusteri@umfst.ro (M.-S.M.); razvan.mares@umfst.ro (R.G.M.); ioan.tilea@umfst.ro (I.T.); 4 Department of Cardiology I, The Emergency Institute for Cardiovascular Diseases and Transplantation, 540136 Targu Mures, Romania; radu.suteu@umfst.ro; 5 Mures County Clinical Hospital, 540072 Targu Mures, Romania; raluca.tilinca@gmail.com

**Keywords:** heart failure, red cell distribution width-to-estimated glomerular filtration rate ratio (RGR), NT-proBNP, risk stratification, mortality

## Abstract

**Background/Objectives:** This study aimed to investigate the prognostic value of the red cell distribution width-to-estimated glomerular filtration rate (RGR) ratio in patients hospitalized with chronic heart failure (CHF) and its potential interaction with NT-proBNP levels. By integrating anemia and renal dysfunction markers, the RGR may provide enhanced predictive insights regarding extended length of hospital stay (ELOS) > 7 days, in-hospital mortality, and 6-month all-cause mortality across specific CHF phenotypes. **Methods:** In this retrospective, single-center pilot observational study, 627 CHF admissions (January 2022–August 2024) were analyzed. Patients were classified according to the ESC guidelines into heart failure with reduced (HFrEF), mildly reduced (HFmrEF), or preserved ejection fraction (HFpEF). The RGR was calculated as red cell distribution width standard deviation (RDW-SD) divided by estimated glomerular filtration rate (eGFR). Predictive accuracy was evaluated using logistic regression, receiver operating characteristic (ROC) analyses, and stepwise Cox proportional hazard regression. **Results:** RGR was significantly higher in HFrEF than in HFpEF (*p* = 0.042) and predicted ELOS only in HFpEF (AUC = 0.619). In contrast, for in-hospital mortality, RGR achieved excellent discrimination in HFrEF (AUC = 0.945), outperforming RDW and NT-proBNP. In HFmrEF, RDW exhibited the highest predictive power (AUC = 0.826), whereas in HFpEF, NT-proBNP was the strongest predictor (AUC = 0.958), although RGR preserved good discrimination (AUC = 0.746). Across the entire cohort and HF phenotypes, RGR consistently emerged as a significant predictor in univariable analysis. In multivariable models, it improved the significance prognosis especially alongside NT-proBNP in the entire cohort and HFrEF. For 6-month all-cause mortality, RGR surpassed RDW in prediction in all HF phenotypes. **Conclusions:** The RGR independently predicts prolonged hospitalization, in-hospital, and 6-month mortality in CHF—often outperforming RDW and eGFR and being comparable to NT-proBNP, especially in HFrEF. These findings suggest that RGR may serve as a valuable risk stratification tool in CHF management.

## 1. Introduction

A 2019 survey by the Heart Failure Association (HFA) of the European Society of Cardiology (ESC) reported a median of >6000 heart failure (HF) hospitalizations per million people in Romania, far exceeding the European median of 2671 cases. This elevated burden may reflect the limited HF-care infrastructure, with fewer than 0.5 dedicated centers per one million inhabitants. Despite high hospitalization rates, the average length of hospital stay (LOS) of 7.3 days for HF in Romania is lower than the European median of 8.5 days, possibly due to overcrowding in the few available HF facilities. Mortality data were not presented, owing to variability in national reporting [1].

The Large-Scale Japanese Registry of Acute Decompensated Heart Failure documented a median LOS of 18 days and an in-hospital mortality of 7.7% [2]. Osenenko et al. reported in a systematic review of HF hospitalizations in the United States (USA) a median for LOS of 3.2–7 days [3]. Comparative analyses further highlighted global discrepancies in LOS, ranging from 4 days (USA) to 7 days (UK), 9 days (Taiwan), and 17 days (Japan). Accordingly, in-hospital mortality was highest in Japan, followed by the UK, Taiwan, and the USA, whereas all-cause 30-day readmissions were most frequent in the USA and least frequent in Japan [4]. Bates et al. similarly reported substantial differences in HF management between the USA and Japan, including greater utilization of cardiovascular medications and higher rates of cardiac rehabilitation at discharge in Japan (31.6% vs. 1.6%) [5]. Smith et al. noted that among HF patients, a primary complaint of pain during emergency medical transport did not increase in-hospital mortality risk, but it was associated with fewer admissions and shorter LOS [6]. Lastly, although longer LOS was observed for Black, Hispanic, and Indigenous American/Pacific Islander patients in the USA, this did not correlate with improved quality of care [7].

The standard deviation of red cell distribution width (RDW-SD) refers to the width of the red blood cell size distribution histogram measured at the 20% height level [8].

The underlying mechanisms linking red cell distribution width (RDW) to heart failure likely involve aging, oxidative stress, systemic inflammation, renal dysfunction, iron deficiency, and nutritional insufficiencies [9]. Felker et al. confirmed RDW as a robust independent indicator of morbidity and mortality using two distinct datasets [10]. Elevated RDW levels have been observed in both HF and non-HF populations, where they remain independently associated with mortality [11]. Although RDW at admission correlates with increased one-year mortality, its predictive power diminishes over longer durations [12].

In patients with suspected HFpEF, RDW provides independent incremental predictive value when combined with myocardial strain assessments via four-dimensional speckle-tracking echocardiography [13]. Notably, Vizzardi et al. demonstrated that RDW is superior to left ventricular mass index and mitral regurgitation grade in predicting outcomes among patients with CHF [14]. Importantly, anemia does not appear to alter RDW levels in HF, and RDW retains its independent predictive strength for mortality and hospitalizations, comparable to N-terminal prohormone of brain natriuretic peptide (NT-proBNP) [15,16]. Additionally, combining hemoglobin with RDW has demonstrated utility in predicting HF-related mortality and cardiovascular hospitalizations [17].

An estimated glomerular filtration rate (eGFR) below 60 mL/min/1.73 m^2^ is independently linked to a higher likelihood of cardiovascular mortality or hospital admission [18,19].

Measurement of NT-proBNP is strongly recommended by current clinical guidelines, reflecting its established prognostic value in heart failure (HF), as was demonstrated in 2003 [20,21].

According to the 2021 ESC Guidelines for the diagnosis and treatment of acute and chronic heart failure, numerous cardiovascular comorbidities are recognized as factors influencing elevated NT-proBNP levels. Additionally, non-cardiac conditions may also affect natriuretic peptide concentrations [20,22]. Notably, paradoxically low NT-proBNP levels have been observed in individuals with obesity [23].

This retrospective observational pilot study distinguishes itself by assessing a newly proposed biomarker—the red cell distribution width-to-estimated glomerular filtration rate (RGR) ratio—and evaluating its prognostic significance in patients with CHF. By integrating the established markers RDW and eGFR, the RGR may offer enhanced prognostic accuracy compared to its individual components. This study, therefore, aims to determine whether this combined biomarker provides superior risk stratification in CHF. Additionally, this research explores whether combining RGR with NT-proBNP, a recognized marker of myocardial stress, further enhances risk stratification for clinical outcomes. Through comprehensive comparative analyses, this investigation examines the individual and combined predictive capacities of RDW, eGFR, NT-proBNP, and RGR in predicting prolonged hospitalization, in-hospital mortality, and 6-month all-cause mortality among patients with CHF, and across different CHF phenotypes. Identifying the most robust prognostic markers could refine current risk stratification methods and significantly advance clinical decision-making in HF management.

## 2. Materials and Methods

This pilot study, designed as a retrospective and observational analysis at a single center, was carried out between 1 January 2022 and 31 August 2024 within the Internal Medicine II—Cardiology Department of the County Emergency Clinical Hospital in Targu Mures, Romania.

Eligible patients had a primary diagnosis of CHF, either previously established or newly identified, based on the 2019 International Classification of Diseases, 10th Revision (ICD-10) discharge codes (I50, I50.1, I50.9). Additional inclusion criteria were classification as New York Heart Association functional class (NYHA-FC) II–IV, a length of hospital stay (LOS) of at least two days, and availability of laboratory data according to the study protocol.

Exclusion criteria included evidence of active inflammation or infection, sepsis, malignancy, autoimmune disease, documented hepatic impairment, neoplasms of the hematologic system (including myelodysplasia), stage five chronic kidney disease (CKD), regardless of dialysis status, and discharge upon patient request.

**Figure 1:**
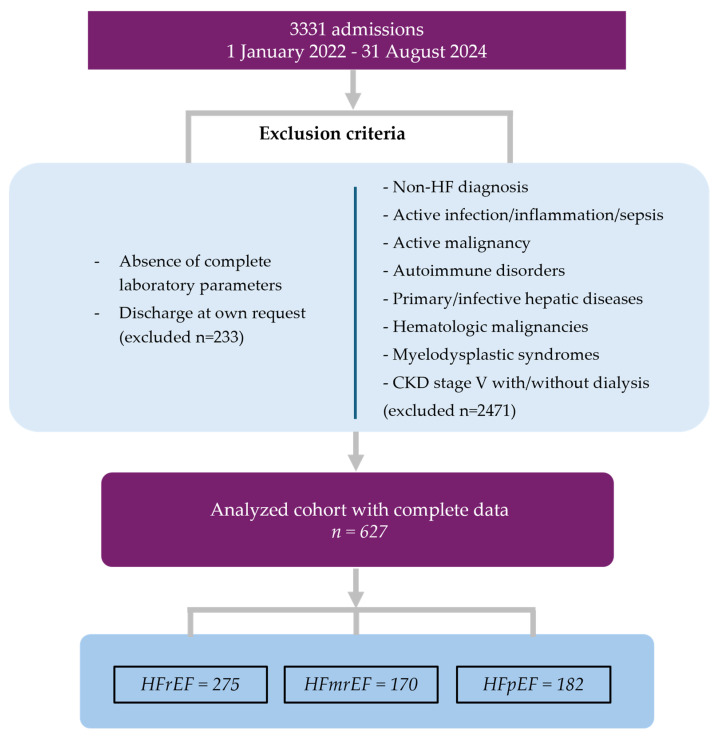
Flow diagram of the studied population. CKD, chronic kidney disease; HF, heart failure; HFmrEF, heart failure with mildly reduced ejection fraction; HFpEF, heart failure with preserved ejection fraction; HFrEF, heart failure with reduced ejection fraction; *n*, number of cases.

Figure 1 presents a flow diagram illustrating the selection of the study population. A total of 627 admissions that met all inclusion and exclusion criteria were enrolled in the study cohort.

HF phenotypes were classified according to the ESC guidelines into HF with reduced ejection fraction (HFrEF), HF with mildly reduced ejection fraction (HFmrEF), and HF with preserved ejection fraction (HFpEF).

Analyzed data included demographic characteristics (age, sex, geographical environment—urban or rural), clinical characteristics (body mass index—BMI, heart rate—HR, systolic and diastolic blood pressure—SBP and DBP, respectively, NYHA-FC), and cardiovascular comorbidities, including documented hypertension, coronary artery disease—CAD, myocardial infarction, heart valve disorders (≥moderate severity), previous heart surgery, atrial fibrillation—AF, and known history of myocarditis. Documented diagnosis of type II diabetes mellitus—T2DM, chronic obstructive pulmonary disease—COPD, low hemoglobin levels—anemia, chronic renal disease (CKD stage I–IV), and thyroid dysfunction were also accounted. A BMI threshold of ≥25 kg/m^2^ was used to define excess body weight in this study.

Atrial fibrillation was evaluated as a component of the heart failure burden, regardless of its temporal pattern or the coexistence of atrial flutter.

Anemia was defined as a cardiovascular comorbidity using a hemoglobin cut-off of <12 g/dL in women and <13 g/dL in men [24].

Hospitalizations lasting longer than seven days were classified as extended length of hospital stay (ELOS).

The initial blood sample, obtained via upper limb venipuncture upon admission, served as the specimen for all laboratory assays. These analyses were conducted in an ISO-15189:2022 [25]-accredited laboratory, ensuring adherence to the highest quality standards set by the International Organization for Standardization.

The diagnostic platforms for laboratory measurements included the Sysmex XN-550 analyzer (Sysmex Corporation, Kobe, Japan) for whole blood, including RDW/RDW-SD, selected to minimize heterogeneity in erythrocyte sizing and RDW values commonly seen due to the lack of harmonization among different analyzer platforms [8]. Biochemical analyses were conducted using the Konelab Prime 60i analyzer (Thermo Fisher Scientific Inc., Waltham, MA, USA), while NT-proBNP levels were measured with the Nano-Checker™ 710 Reader (Nano-Ditech Corporation, Cranbury, NJ, USA).

Among hematological indices, RDW-SD was registered. The biochemical assessments included serum creatinine and eGFR, while cardiac stress was evaluated based on circulating NT-proBNP levels. The eGFR (mL/min/1.73 m^2^) was calculated with the 2021 CKD-EPI formula.

Heart failure treatment was in-line with the guidelines at the time of this study. Patients were discharged upon clinical improvement, and six-month all-cause mortality was ascertained through telephone follow-up.

### 2.1. Red Cell Distribution Width-to-Estimated Glomerular Filtration Rate Ratio (RGR)

A decline in erythropoietin production contributes to the development of anemia and enhances heterogeneity in erythrocyte morphology, as reflected by elevated RDW. Consequently, the impact of renal function on RDW remains a critical factor in shaping this hematological parameter.

The RGR was determined by taking the ratio of RDW-SD to eGFR, as follows:

$$RGR=\frac{RDW-SD(fl)}{eGFR(mL/min/1.73m²)}$$

The ratio was treated as dimensionless in the statistical analysis.

### 2.2. Statistical Analysis

MedCalc^®^ Statistical Software version 23.1.6 (MedCalc Software Ltd., Ostend, Belgium; https://www.medcalc.org; 2025, last accessed on 20 February 2025) was used for data analysis. A *p*-value of ≤0.05 was considered statistically significant.

Normality of distribution was assessed using the Kolmogorov–Smirnov test. Outliers were not excluded on the assumption of valid biological variability [26]. All continuous variables (age, BMI, HR, SBP, DBP, total number of comorbidities, LOS) deviated from normal distribution; therefore, continuous data were presented as median and interquartile ranges (IQR). Categorical variables, including cardiovascular comorbidities, in-hospital mortality, 6-month all-cause mortality, and overall mortality, were reported as frequencies and percentages. The NYHA-FC was recorded categorically, with NYHA-FC III and IV grouped together for the Cox proportional hazards analysis (NYHA-FC II = 0; NYHA-FC III and IV = 1). Due to NT-proBNP’s high biological variability, we applied a base-10 logarithmic transformation for both uni- and multivariable analyses.

Potential cofounders were analyzed among the studied parameters.

For comparisons across HF phenotypes, the Kruskal–Wallis test was applied, and when appropriate, followed by Dunn’s post-hoc test [27,28]. Statistical differences between variables were also evaluated with the Mann–Whitney U-Test and chi-square test (χ^2^-value) when appropriate. Cramér’s V test was used to assess the effect size.

Univariate logistic regression was used to assess the independent associations between potential predictors and extended hospitalization. For in-hospital mortality, stepwise Cox proportional hazards regression (both univariate and multivariate) was applied to determine independent predictors. Harrell’s C-index (with 95% CI) was calculated to evaluate the model’s goodness of fit.

Prediction models derived from the univariable analyses were compared using the DeLong test, following receiver operating characteristic (ROC) analysis to obtain the area under the curve (AUC) [29]. Due to the extensive amount of data presented, only ROC curves and the key observations are included here; differences between areas, 95% CIs, and *p*-values were omitted in the interest of brevity. The Youden index was used to identify the optimal threshold for the markers of interest, thereby maximizing diagnostic effectiveness [30].

## 3. Results

### 3.1. Study Population, Demographic and Clinical Profile

The study cohort included 627 hospital admissions related to CHF that were analyzed, with a median age of 71 years (IQR: 62–77). Significant age differences were observed among HF phenotypes (*p* = 0.004), particularly between HFrEF and HFpEF, as shown by post-hoc tests. Male patients comprised 55.02% of the cohort and were more prevalent in the HFrEF and HFmrEF phenotypes. A chi-square test revealed a statistically significant association between gender and HF phenotype (χ^2^(2) = 22.33, *p* < 0.001, Cramér’s V = 0.19).

Although most patients were residents in urban areas, no significant correlation was found between environmental background and HF phenotype (χ^2^(4) = 4.28, *p* = 0.369, Cramér’s V = 0.06).

The mean BMI of the patients was 28.41 kg/m^2^, indicative of excess body weight, with no significant variation among HF phenotypes (*p* = 0.234).

An overview of the main characteristics of the entire cohort and each HF phenotype is summarized in Table 1.

Vital signs (HR, SBP, and DBP) were assessed. Patients with HFrEF exhibited a significantly higher HR compared to those with HFmrEF or HFpEF (post-hoc test, *p* < 0.001). In contrast, SBP values at admission were significantly raised in HFpEF relative to HFrEF (*p* < 0.001), while DBP values did not differ significantly among the groups.

Two-thirds of patients were classified as NYHA-FC III or IV; HFpEF was more frequently associated with NYHA-FC II. The chi-square test described a significant association between NYHA-FC and HF phenotypes (χ^2^(4) = 58.06, *p* < 0.001, Cramér’s V = 0.22).

One in two patients presented at least five comorbidities. The total number of comorbidities differed significantly across NYHA-FC (*p* = 0.003); NYHA-FC II patients had fewer comorbidities than those in NYHA-FC III or IV (post-hoc analysis). No significant differences were identified regarding geographical background or HF phenotype (*p* > 0.05) regarding the total number of comorbidities.

Excess body weight was similarly distributed among HF phenotypes. The two most prevalent comorbidities were hypertension (82.46%) and moderate or severe valvular heart disease (81.66%). The Chi-square test revealed noteworthy expected frequencies for CAD, prior myocardial infarction, hypertension, prior valvular surgery, COPD, and dysthyroidism.

### 3.2. Length of Hospital Stay

The full cohort had a median length of hospital stay of 7 days, with no statistically significant variation between the different HF phenotypes (*p* = 0.111). ELOS was recorded in 43.48% of cases and showed a significant association with HF phenotype (χ^2^(2) = 8.93, *p* = 0.011, Cramér’s V = 0.12). However, the overall hospitalization duration did not significantly differ (*p* = 0.945) among those with ELOS. Patients with ELOS exhibited a significantly higher total number of comorbidities (median of five, IQR 4–6, *p* = 0.038) and were classified within a more severe NYHA functional class (*p* = 0.003) compared to those with LOS ≤ 7 days.

### 3.3. Laboratory Data

Table 2 depicts the laboratory data for the entire cohort and each HF phenotype. RDW did not differ significantly among HF phenotypes. In contrast, post-hoc analysis showed significantly higher creatinine in HFrEF patients compared to HFpEF cases, indicating greater renal impairment in patients with reduced LVEF. eGFR levels did not differ significantly among the HF phenotypes (*p* = 0.056).

RGR values were evaluated for the entire cohort. Elevated RGR levels were associated with ELOS, valvular heart disease ≥ moderate, AF, T2DM, anemia, and pre-existing CKD. There was a slight positive correlation between RGR and the overall burden of comorbidities (r = 0.18, *p* < 0.001). Furthermore, RGR values differed significantly across HF phenotypes (*p* = 0.042), with post-hoc analyses indicating higher values in HFrEF compared to HFpEF.

Additionally, the degree of myocardial stress, quantified by NT-proBNP, aligned with HF severity (*p* < 0.001), such that more advanced HF phenotypes exhibited higher NT-proBNP levels.

### 3.4. Predictors of Prolonged Hospitalization

The results of a univariate analysis conducted to identify predictors of ELOS are presented in Table 3.

For the entire cohort, NT-proBNP demonstrated the highest AUC (0.647); RDW ranked second with an AUC of 0.603 (*p* < 0.001).

An analysis by HF phenotype demonstrated that in HFrEF, both NT-proBNP and RDW significantly predicted ELOS, with AUC values of 0.623 and 0.620, in that order (*p* < 0.001). In the HFmrEF phenotype, RDW exhibited a reduced predictive value, leaving NT-proBNP as the only marker with sufficient discriminative power for ELOS. In HFpEF, although RGR yielded an AUC of 0.619 (*p* < 0.001), NT-proBNP remained the strongest predictor of prolonged hospitalization, with an AUC of 0.628.

**Figure 2:**
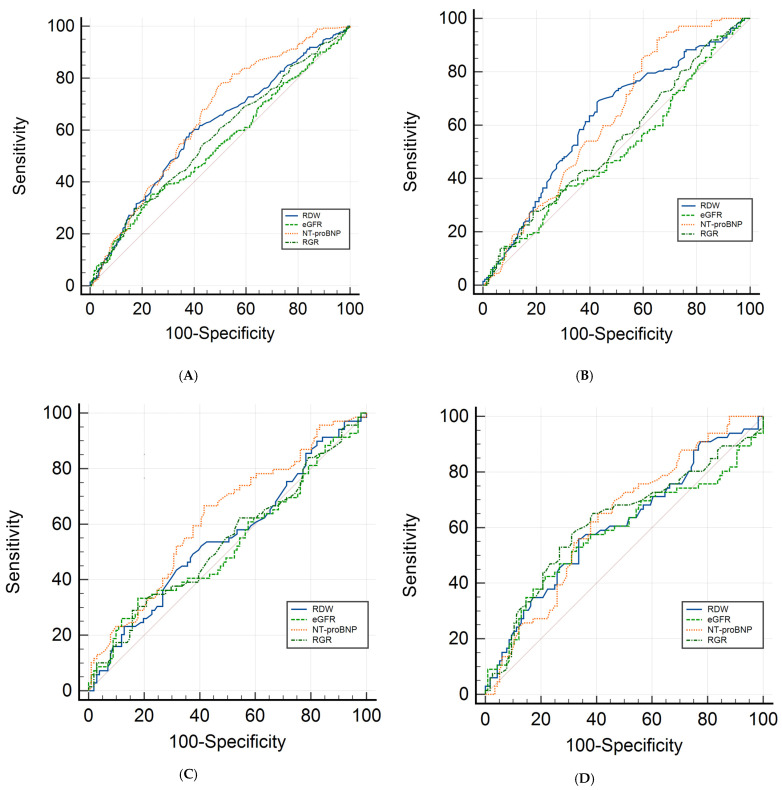
Comparison of ROC curves for predicting ELOS in: (**A**) the entire cohort, (**B**) HFrEF phenotype, (**C**) HFmrEF phenotype, and (**D**) HFpEF phenotype. eGFR, estimated glomerular filtration rate; RDW, red cell distribution width; RGR, red cell distribution width-to-estimated glomerular filtration rate ratio.

Differences in discriminatory power were assessed using ROC curve comparisons (Figure 2). In the overall cohort (quadrant A, top-left), NT-proBNP and RDW exhibited comparable predictive performance (difference between areas = 0.044, 95% CI: −0.006 to 0.095, *p* = 0.087), while both significantly outperformed eGFR and RGR.

In the HFrEF phenotype (quadrant B, top-right), a similar pattern emerged, with NT-proBNP and RDW serving as the primary predictors without a significant difference between them.

In the HFmrEF phenotype (quadrant C, bottom-left), only NT-proBNP demonstrated a statistically significant AUC (*p* < 0.05).

Within the HFpEF phenotype (quadrant D, bottom-right), both NT-proBNP and RGR demonstrated reliable predictive capacity for ELOS, with no statistically significant difference observed between their respective AUC values. Importantly, RGR exhibited superior predictive performance compared to eGFR in this specific phenotype.

### 3.5. Predictors of In-Hospital Mortality

Regarding in-hospital mortality (Table 4), all biomarkers demonstrated markedly superior predictive performance compared to their ELOS predictions. For the overall cohort, RGR, NT-proBNP, and eGFR achieved excellent discrimination, with AUC values ranging from 0.806 to 0.830, while RDW exhibited robust predictive capability (AUC = 0.741).

Within the HFrEF cohort, biomarkers demonstrated notably robust predictive capabilities. Both RGR and eGFR achieved excellent discrimination, with AUCs of 0.945 and 0.919, respectively, while NT-proBNP exhibited very good performance (AUC = 0.822). In contrast, RDW yielded good predictive accuracy with an AUC of 0.727.

In the HFmrEF cases, RDW attained the highest AUC at 0.826, reflecting very good discrimination, whereas NT-proBNP, eGFR, and RGR showed good predictive power, with AUC values ranging from 0.715 to 0.769.

Finally, among HFpEF patients, NT-proBNP emerged as the strongest predictor of in-hospital mortality, achieving an excellent AUC of 0.958 (*p* < 0.001). This was followed by eGFR and RGR, which both demonstrated good discrimination, while RDW maintained sufficient predictive capacity.

**Figure 3:**
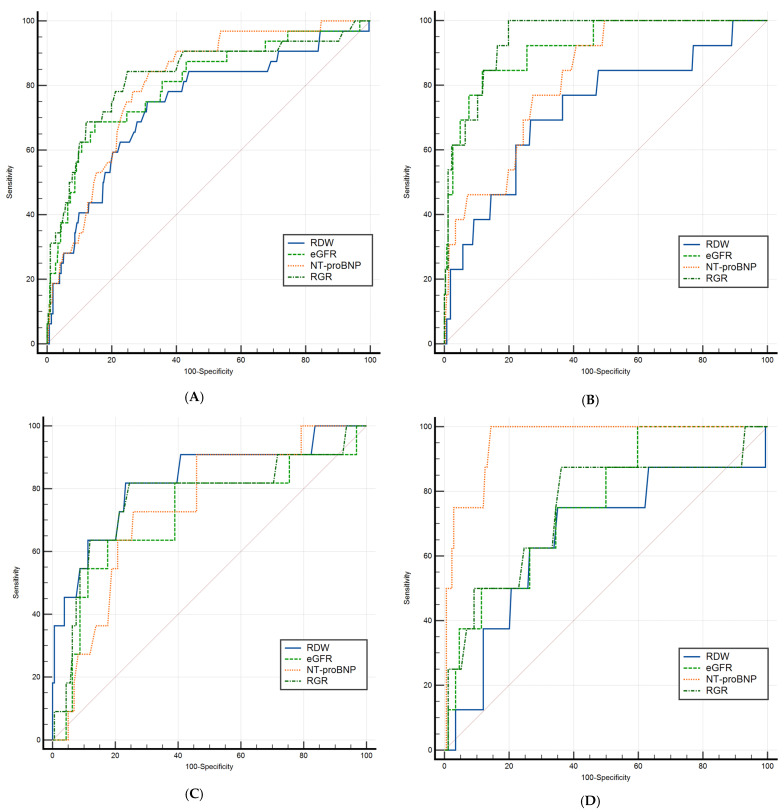
ROC curve comparison predicting in-hospital mortality for: (**A**) entire cohort, (**B**) HFrEF, (**C**) HFmrEF, and (**D**) HFpEF. eGFR, estimated glomerular filtration rate; NT-proBNP, N-terminal prohormone of brain natriuretic peptide; RDW, red cell distribution width; RGR, red cell distribution width-to-estimated glomerular filtration rate ratio.

An ROC curve comparison analysis was subsequently conducted (Figure 3).

For the entire cohort (quadrant A, top-left), ROC curve comparisons revealed no significant differences among the predictive models. Within the HFrEF cases (quadrant B, top-right), RGR had a statistically significantly higher AUC than RDW and NT-proBNP and exceeded eGFR’s AUC numerically, although the latter difference was not statistically significant.

In HFmrEF patients (quadrant C, bottom-left), only one significant difference emerged among the four biomarkers, namely, between RGR and eGFR (difference between areas = 0.054, 95% CI: 0.011–0.097, *p* = 0.012).

Within the HFpEF cases (quadrant D, bottom-right), NT-proBNP was the strongest predictor, outperforming all three other biomarkers, while RDW, eGFR, and RGR did not significantly differ from one another.

### 3.6. Cox Proportional Hazards Regression for In-Hospital Mortality

Several factors were evaluated for their association with in-hospital mortality. Data are presented in Table 5.

Cox proportional hazards univariate regression was performed on each parameter for the overall cohort and separately by HF phenotype. For parameters reaching statistical significance (*p* ≤ 0.05), Harrell’s C-index was calculated to assess discriminatory ability.

Age emphasized an effect on mortality risk in the entire cohort, but exhibited poor discriminatory power. Within the HF phenotypes, age did not achieve statistical significance. However, risk over-time expresses an exponential power with age advancement; for example, in ten years, relative risk is 1.036^(10)^ = 1.423.

Within the entire cohort, an elevated heart rate was significantly associated with increased mortality risk (hazard ratio = 1.015, 95% CI: 1.002–1.028, *p* = 0.028), demonstrating nearly acceptable discrimination. However, this association was not observed when analyzing individual HF phenotypes. Consequently, substantial variations in heart rate exert a pronounced effect on relative risk, with each unit increase corresponding to a 1.015-fold rise in risk.

Elevated SBP emerged as a protective factor, demonstrating moderate discriminatory capacity across the entire cohort. This effect was particularly pronounced in HFrEF patients—where cardiac pump function is significantly impaired—and was supported by a robust Harrell’s C-index of 0.765. Similarly, higher DBP values were associated with reduced mortality risk, representing a modest yet statistically significant protective factor with good discriminatory power. This association persisted among both HFrEF and HFmrEF patients. The hazard ratio is interpreted similarly to that for heart rate, indicating that each incremental increase in SBP or DBP confers an enhanced protective effect.

In this study, advanced NYHA-FC (IV) correlated with an increased risk of mortality and demonstrated sufficient discrimination in the entire cohort, and good and very good discrimination in HFrEF and HFpEF, respectively. The HFmrEF patients did not show a significant relative risk, although further investigation in a larger cohort may clarify this finding.

The total number of comorbidities emerged as a key predictor only in the HFrEF phenotype. This must be interpreted with caution since only 13 known comorbidities were considered as equal contributing effect, and the severity of each comorbidity was not assessed. An additional comparison regarding the total number of comorbidities showed no significant difference between deceased and discharged patients (*p* = 0.118).

RDW emerged as a robust predictor of mortality risk across the entire cohort, demonstrating strong discriminatory performance in both HFrEF and HFmrEF cases. Renal function, as indicated by higher eGFR values, conferred an overall protective effect and exhibited good to excellent discrimination in the HFrEF and HFpEF subgroups. Furthermore, NT-proBNP consistently showed good to excellent predictive value for in-hospital mortality across the overall cohort, as well as within the HFrEF and HFpEF populations.

Finally, RGR emerged as a strong discriminative predictor for the entire cohort (hazard ratio: 1.703, 95% CI: 1.485–1.953) and maintained its significance across all HF phenotypes, showing excellent to good discrimination, consistent with its values observed in Table 2.

To delineate the combined key risk factors for in-hospital mortality across the entire cohort and within specific HF phenotypes, Cox proportional hazards models were applied using a stepwise selection method, as summarized in Table 6. For the overall cohort, age, NYHA-FC, NT-proBNP, and RGR collectively provided robust discriminatory power.

In the HFrEF subgroup, the total number of comorbidities, NT-proBNP, and RGR were significant contributors to mortality risk.

Conversely, in the HFmrEF subgroup, RDW emerged as the predominant predictor, underscoring the critical impact of anemia. Lastly, in the HFpEF subgroup, NYHA-FC at admission and NT-proBNP were identified as the primary risk indicators.

### 3.7. Predictors of 6-Month All-Cause Mortality

In the analysis for 6-month all-cause mortality, all four biomarkers (RDW, eGFR, NT-proBNP, and RGR) demonstrated sufficient to good AUC values (Table 7). The sole exception was RDW in the HFpEF subgroup, which showed no discriminatory power.

**Figure 4:**
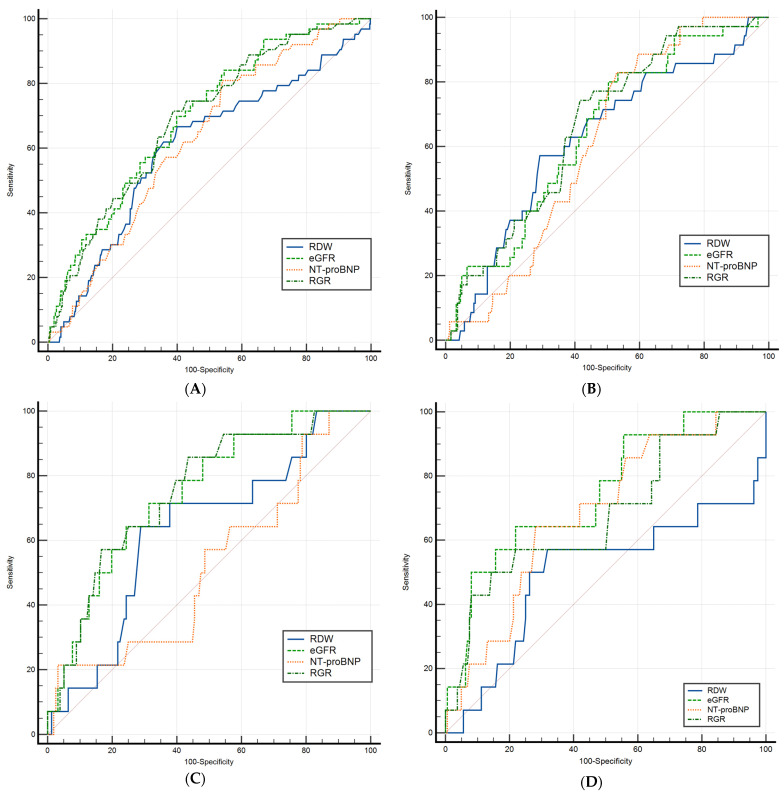
ROC curve comparisons for predicting 6-month all-cause mortality across: (**A**) the entire cohort, (**B**) HFrEF, (**C**) HFmrEF, and (**D**) HFpEF. eGFR, estimated glomerular filtration rate; NT-proBNP, N-terminal prohormone of brain natriuretic peptide; RDW, red cell distribution width; RGR, red cell distribution width-to-estimated glomerular filtration rate ratio.

ROC curve comparison was applied for 6-month all-cause mortality (Figure 4).

In the overall cohort (quadrant A, top-left), RGR and eGFR demonstrated superior predictive performance relative to RDW, yet neither differed significantly from NT-proBNP or from one another.

Within the HFrEF cases (quadrant B, top-right), no significant differences emerged among the four biomarkers.

In the HFmrEF phenotype (quadrant C, bottom-left), both the renal–anemia axis (RGR) and eGFR exceeded NT-proBNP in predictive power, with no further significant AUC differences detected.

Ultimately, in the HFpEF subgroup (quadrant D, bottom-right), eGFR, NT-proBNP, and RGR each exhibited moderate to good discrimination, all significantly outperforming RDW while remaining statistically indistinguishable from one another in terms of predictive ability.

## 4. Discussions

This pilot study provides novel insights into the prognostic utility of the RGR in CHF, further compared with the interplay with RDW, eGFR, and NT-proBNP. Our results highlight the independent predictive value of RGR for prolonged hospitalization and in-hospital mortality, emphasizing its potential role as a composite biomarker integrating hematologic and renal dysfunction. The observed correlation further supports the notion that integrating renal, hematologic, and cardiac biomarkers may enhance risk stratification in CHF.

In our study, RDW demonstrated strong short-term prognostic performance for in-hospital mortality, and its predictive power started diminishing on long-term. This is similar to previous studies [12]. Muhlestein et al. reported that baseline RDW values and changes in RDW during hospitalizations were linked with longer LOS, first-month all-cause readmission, and mortality [31]. This observation is preserved and available in patients with acute HF as well [32]. A meta-analysis of multiple studies conducted by Huang et al. further confirmed the association between elevated RDW values and poorer prognosis in heart failure [33]. Notably, one study found that RDW can independently predict adverse outcomes, separate from NT-proBNP’s prognostic influence [34].

NT-proBNP was a robust predictor of adverse outcomes in our retrospective pilot study, aligning with previous findings of Gardner et al. [21].

However, our research demonstrated that RGR, a novel composite parameter, provides additional prognostic value across different CHF phenotypes, particularly in HFrEF. The superior performance of RGR over RDW and eGFR in mortality prediction suggests that it effectively captures the interplay between anemia, renal impairment, and myocardial stress, three key determinants of HF progression.

Stratified analyses indicated that in HFrEF patients, both NT-proBNP and RGR strongly predicted in-hospital mortality, with RGR outperforming eGFR—a finding that underscores the known link between renal dysfunction and adverse outcomes in this phenotype. In HFmrEF, RDW emerged as the most powerful predictor, highlighting the role of anemia-related mechanisms. Meanwhile, in HFpEF, NT-proBNP remained the most reliable marker, although RGR and eGFR also contributed significant prognostic value, reflecting the impact of renal dysfunction. These differences among HF phenotypes emphasize the need to address all comorbidities in heart failure, rather than focusing solely on cardiovascular factors.

Cox regression analyses (univariable and multivariable; Table 5 and Table 6) revealed that combining RGR and NT-proBNP enhanced prognostic accuracy, as shown by a higher Harrell’s C-index—especially in HFrEF patients—compared to using either marker alone. This emphasized the interconnection and role of these two biomarkers and their involvement in prognosis prediction. Furthermore, in HFmrEF, RGR’s C-index, although lower, was comparable to that of RDW, while in HFpEF, NT-proBNP and advanced NYHA class carried a significantly higher risk than RGR.

Importantly, RGR’s predictive strength for mortality matched that of guideline-endorsed NT-proBNP, underscoring its clinical value. Given the routine availability of RDW and eGFR, the RGR may serve as a cost-effective tool for early risk stratification in CHF. Finally, the diminished discriminatory power at the 6-month all-cause mortality endpoint suggests that while biomarkers are vital for short-term prognosis, additional factors likely influence long-term outcomes.

In the Cox regression univariate analysis, the prognostic roles of age, HR, SBP, and DBP were consistent with findings from prior HF studies [35,36,37,38], while contributing a role in the relative risk for the total number of comorbidities in HFrEF both in uni- and multivariate analysis Cox regression. Notably, while most literature evaluates comorbidities across HF irrespective of phenotype, our findings specifically underscore their impact in the HFrEF subgroup [39,40].

Given that RDW and eGFR are routinely available upon admission, the RGR ratio can be easily calculated at the bedside or integrated into electronic health systems to support real-time risk stratification. In our study, RGR showed strong prognostic value for in-hospital mortality, especially in patients with HFrEF, and may be particularly useful in clinical settings where NT-proBNP is unavailable or difficult to interpret due to comorbid conditions such as obesity or renal impairment. Moreover, while our analysis focused on single-point measurements at admission, future prospective studies should explore validating these findings in larger multicentric cohorts, assessing whether dynamic changes in RDW, eGFR, and NT-proBNP over time—and consequently in RGR—might enhance prognostic accuracy and better reflect clinical evolution throughout hospitalization or follow-up.

A promising future avenue of research involving RDW and RGR could be the assessment of sleep apnea as a frequent coexisting comorbidity, given its known associations with both cardiovascular and hematologic alterations [41].

### Study Limitations

Although this is a pilot study which focuses on formulation of some new hypotheses for future main studies, several limitations should be acknowledged. First, its single-center design may limit the generalizability of findings to broader populations, necessitating external validation in multicenter cohorts. Second, the retrospective nature of the analysis introduces potential selection bias and limits causal inferences. Third, while the study provides novel insights into the prognostic utility of the RGR in CHF, further research is required to confirm its predictive value across diverse healthcare settings.

Patients with stage 5 CKD and those with active inflammatory, infectious, or autoimmune conditions were excluded, as these comorbidities are known to significantly affect RDW and eGFR values independently of cardiovascular status. In advanced renal failure, chronic inflammation, erythropoietin resistance, and metabolic dysregulation may confound the interpretation of RDW [42,43]. Similarly, systemic inflammation or infection can elevate RDW irrespective of HF severity, limiting its prognostic specificity [44].

Additionally, long-term follow-up was limited to six months, leaving the impact of RGR on extended survival outcomes undetermined. The reliance on a single admission biomarker value for RDW, estimated eGFR, and NT-proBNP does not account for dynamic changes over time, which may have clinical relevance. Furthermore, potential confounding variables—such as socioeconomic factors or frailty—were not fully accounted for, despite multivariable adjustments.

The assessment of anemia and renal dysfunction severity was limited, as additional markers such as ferritin, transferrin saturation, and proteinuria were not included in the analysis. Therapeutic stratification was not performed, meaning the potential influence of CHF treatment variations, including guideline-directed medical therapy and cardiac rehabilitation, remains unknown.

An important limitation relates to using eGFR as a surrogate for actual renal function. All widely adopted formulas, including CKD-EPI, are based on serum creatinine, a biomarker whose concentration is subject to considerable variability due to age, muscle mass, and comorbid conditions. In elderly individuals or patients with reduced muscle mass, the discordance between eGFR and directly measured GFR can be clinically meaningful, resulting in either overestimation or underestimation of renal function. This is particularly pertinent in our study, where RGR incorporates eGFR in its denominator. Consequently, any intrinsic imprecision in the estimation of GFR has the potential to compromise both the reliability and interpretability of RGR, especially in populations with atypical creatinine kinetics. Future investigations should prioritize the inclusion of measured GFR, where feasible, or consider complementary biomarkers such as cystatin C, to improve the accuracy and applicability of RGR across heterogeneous patient populations.

Lastly, although comorbidities were analyzed, their real cumulative impact on patient prognosis remains incompletely understood and a complex challenge. Future prospective, multicenter studies with extended follow-up and comprehensive therapeutic profiling are warranted to further validate RGR as a prognostic biomarker in CHF or possible other pathologies and assess its role in clinical decision-making.

## 5. Conclusions

This study highlights the prognostic significance of RGR in CHF, demonstrating its independent predictive value for prolonged hospitalization, in-hospital death, and all-cause mortality at six months. RGR consistently outperformed or was on equal par with its individual components (RDW and eGFR) in most scenarios and outcomes and it exhibited comparable predictive power to NT-proBNP, particularly in the entire cohort and in the subgroup of patients with HFrEF.

Given its accessibility, cost-effectiveness, and ease of calculation from routinely available laboratory parameters, RGR may serve as a valuable biomarker for early risk stratification in CHF. Its integration into current HF prognostic models could refine risk assessment, aiding in clinical decision-making and optimizing patient management. However, further prospective, multicenter studies are needed to confirm its clinical utility and to elucidate its potential role in guiding therapeutic strategies for heart failure.

## Figures and Tables

**Table 1:** Characteristics of the entire studied cohort and each HF phenotype.

Parameter	Entire Cohort n = 627	HFrEF n = 275	HFmrEF n = 170	HFpEF n = 182	*p* Value
Age (years, median)	71 (62–77)	70 (59–77)	71 (63–77.75)	73 (66.25–78.75)	0.004 *
Male (n, %)	345 (55.02)	175 (63.63)	95 (55.88)	75 (41.20)	<0.001 **
Urban areas (n, %)	394 (62.83)	174 (63.27)	114 (67.06)	106 (58.24)	0.369 **
Excess body weight (kg/m^2^, median)	28.41 (24.84–33)	27.68 (24.75–32.08)	29.3 (25.64–32.83)	29.33 (24.57–34.1)	0.234 *
**Clinical characteristics**					
HR (bpm, median, IQR)	77 (67–92)	82 (70–100)	72 (63.5–87.75)	74 (65–85)	<0.001 *
SBP (mmHg, median, IQR)	130 (120–140)	125 (110–140)	130 (120–140)	135 (125–140)	<0.001 *
DBP (mmHg, median, IQR)	80 (70–85)	80 (70–89.5)	75 (70–85)	80 (70–88.75)	0.319 *
NYHA - FC					<0.001 **
II	203 (32.38)	58 (21.09)	60 (35.29)	85 (46.7)	-
III	355 (56.62)	164 (59.64)	105 (61.76)	86 (47.25)	-
IV	69 (11)	53 (19.27)	5 (2.94)	11 (6.04)	-
**Comorbidities**					
Total number (median)	5 (4–6)	5 (4–6)	5 (4–6)	5 (4–6)	0.337 *
Excess body weight (n, %)	460 (73.37)	198 (43.04)	132 (28.7)	130 (28.26)	0.332 **
CAD (n, %)	303 (48.33)	136 (49.45)	92 (54.12)	75 (41.21)	0.047 **
Prior documented MI (n, %)	153 (24.4)	90 (32.73)	39 (22.94)	24 (13.19)	<0.001 **
Hypertension (n, %)	517 (82.46)	207 (75.27)	150 (88.24)	160 (87.91)	<0.001 **
Valvular heart disease ≥ moderate (n, %)	512 (81.66)	236 (85.82)	135 (79.41)	141 (77.47)	0.053 **
Prior valvular surgery (n, %)	33 (5.26)	9 (3.27)	8 (4.71)	16 (8.79)	0.033 **
AF (n, %)	285 (45.45)	129 (46.91)	68 (40)	88 (48.35)	0.236 **
History of myocarditis (n, %)	11 (1.75)	7 (2.55)	4 (2.35)	0 (0)	0.100 **
T2DM (n, %)	221 (35.25)	95 (34.55)	58 (34.12)	68 (37.36)	0.774 **
COPD (n, %)	95 (15.15)	48 (17.45)	33 (19.41)	14 (7.69)	0.003 **
Anemia (n, %)	170 (27.11)	71 (25.82)	49 (28.82)	50 (27.47)	0.780 **
Prior documented CKD (n, %)	185 (29.51)	80 (29.09)	54 (31.76)	51 (28.02)	0.729 **
Dysthyroidism (n, %)	113 (18.02)	42 (15.27)	27 (15.88)	44 (24.18)	0.037 **
**Length of hospital stay**					
LOS (days, median)	7 (5–10)	7 (5–10.5)	7 (5–9)	7 (5–9)	0.111 *
ELOS (n, %)	272 (43.38)	137 (49.82)	69 (40.59)	66 (36.26)	0.011 **
ELOS (days, median)	11 (9–14)	11 (9–13)	10 (8–13)	12 (9–14)	0.945 *
**Mortality**					
Overall mortality	95 (15.15)	49 (17.45)	25 (14.70)	23 (12.08)	0.288 **
In-hospital mortality (n, %)	32 (5.1)	13 (4.73)	11 (6.47)	8 (4.40)	0.630 **
6-months mortality (n, %)	63 (10.04)	35 (12.72)	14 (8.23)	14 (7.69)	0.141 **

AF, atrial fibrillation/atrial flutter; bpm, beats per minute; CAD, coronary artery disease; CKD, chronic kidney disease; COPD, chronic obstructive pulmonary disease; DBP, diastolic blood pressure; ELOS, extended length of hospital stay, HF, heart failure; HFmrEF, heart failure with mildly reduced ejection fraction; HFpEF, heart failure with preserved ejection fraction; HFrEF, heart failure with reduced ejection fraction; HR, heart rate; LOS, length of hospital stay; n, number of cases; MI, myocardial infarction; NYHA-FC, New York Heart Association functional class; SBP, systolic blood pressure; T2DM, type 2 diabetes mellitus. *p* values determined across HF phenotypes, * Kruskal–Wallis test, ** chi-square test.

**Table 2:** Laboratory data for the entire cohort and HF phenotypes.

Parameter	Entire Cohort n = 627	HFrEF n = 275	HFmrEF n = 170	HFpEF n = 182	*p* Value
RDW (fl)	46.3 (43.1–50.4)	46.8 (43.9–51.75)	46.05 (43–49.43)	45.7 (42.03–50.23)	0.112 *
Creatinine (mg/dL)	1.01 (0.8–1.29)	1.05 (0.84–1.33)	1.02 (0.82–1.29)	0.94 (0.71–1.2)	0.004 *
eGFR (mL/min/1.73 m^2^)	73.68 (54.75–97.09)	71.56 (54.66–92.25)	71.63 (52.87–95.87)	78.24 (58.19–102.59)	0.056 *
RGR	0.64 (0.47–0.88)	0.66 (0.5–0.9)	0.64 (0.47–0.89)	0.58 (0.43–0.85)	0.042 *
NT-proBNP (pg/mL)	3.46 (2.82–3.81)	3.73 (3.39–4.02)	3.36 (2.66–3.76)	2.98 (2.59–3.51)	<0.001 *

eGFR, estimated glomerular filtration rate; HFmrEF, heart failure with mildly reduced ejection fraction; HFpEF, heart failure with preserved ejection fraction; HFrEF, heart failure with reduced ejection fraction; n, number of cases; NT-proBNP, N-terminal prohormone of brain natriuretic peptide; RDW, red cell distribution width; RGR, red cell distribution width-to-estimated glomerular filtration rate ratio. * Kruskal–Wallis test.

**Table 3:** Predictors of prolonged hospitalization across entire cohort and HF phenotypes.

Parameter	Dependent Expected Value (ELOS)		
Entire Cohort (n = 627)	AUC (95% CI)	*p* Value	Cut-Off Point
RDW	0.603 (0.563–0.641)	<0.001	>46.6
eGFR	0.539 (0.499–0.579)	0.093	≤57.5
RGR	0.570 (0.530–0.609)	0.002	>0.92
NT-proBNP	0.647 (0.608–0.685)	<0.001	>3.2
**HFrEF (n = 275)**			
RDW	0.620 (0.560–0.678)	<0.001	>45.8
eGFR	0.503 (0.443–0.564)	0.927	≤81.89
RGR	0.553 (0.450–0.653)	0.364	>0.55
NT-proBNP	0.623 (0.563–0.680)	<0.001	>3.79
**HFmrEF (n = 170)**			
RDW	0.548 (0.470–0.624)	0.291	>47.8
eGFR	0.525 (0.447–0.602)	0.586	≤50.41
NT-proBNP	0.624 (0.547–0.697)	0.004	>3.32
RGR	0.537 (0.459–0.614)	0.422	>1.01
**HFpEF (n = 182)**			
RDW	0.606 (0.531–0.678)	0.167	>46.8
eGFR	0.581 (0.506–0.653)	0.087	≤61.95
RGR	0.619 (0.545–0.690)	0.008	>0.65
NT-proBNP	0.628 (0.553–0.698)	0.002	>2.99

AUC, area under the curve; CI, confidence interval; eGFR, estimated glomerular filtration rate; ELOS, extended length of hospital stay; HFmrEF, heart failure with mildly reduced ejection fraction; HFpEF, heart failure with preserved ejection fraction; HFrEF, heart failure with reduced ejection fraction; n, number of cases; NT-proBNP, N-terminal prohormone of brain natriuretic peptide; RDW, red cell distribution width; RGR, red cell distribution width-to-estimated glomerular filtration rate ratio.

**Table 4:** Predictors of in-hospital mortality across entire cohort and HF phenotypes.

Parameter	Dependent Expected Value (In-Hospital Mortality)		
Entire Cohort (n = 627)	AUC (95% CI)	*p* Value	Cut-Off Point
RDW	0.741 (0.705–0.775)	<0.001	>48.7
eGFR	0.806 (0.773–0.836)	<0.001	≤46.84
RGR	0.830 (0.798–0.859)	<0.001	>0.84
NT-proBNP	0.821 (0.771–0.865)	<0.001	>3.79
**HFrEF (n = 275)**			
RDW	0.727 (0.671–0.779)	0.004	>50.4
eGFR	0.919 (0.881–0.949)	<0.001	≤45
RGR	0.945 (0.910–0.968)	<0.001	>0.91
NT-proBNP	0.822 (0.772–0.866)	<0.001	>3.79
**HFmrEF (n = 170)**			
RDW	0.826 (0.760–0.880)	<0.001	>48.8
eGFR	0.715 (0.641–0.781)	0.026	≤46.84
RGR	0.769 (0.699–0.830)	0.003	>0.85
NT-proBNP	0.740 (0.667–0.804)	<0.001	>3.72
**HFpEF (n = 182)**			
RDW	0.662 (0.588–0.730)	0.161	>47.6
eGFR	0.761 (0.692–0.821)	0.001	≤70.09
RGR	0.746 (0.676–0.808)	0.023	>0.67
NT-proBNP	0.958 (0.918–0.982)	<0.001	>3.58

AUC, area under the curve; CI, confidence interval; eGFR, estimated glomerular filtration rate; HFmrEF, heart failure with mildly reduced ejection fraction; HFpEF, heart failure with preserved ejection fraction; HFrEF, heart failure with reduced ejection fraction; n, number of cases; NT-proBNP, N-terminal prohormone of brain natriuretic peptide; RDW, red cell distribution width; RGR, red cell distribution width-to-estimated glomerular filtration rate ratio.

**Table 5:** Cox proportional hazards regression for in-hospital mortality across entire cohort and HF phenotypes.

Parameter	Entire Cohort n = 627		HFrEF n = 275		HFmrEF n = 170		HFpEF n = 182	
	Hazard Ratio (95% CI)	*p* Value	Hazard Ratio (95% CI)	*p* Value	Hazard Ratio (95% CI)	*p* Value	Hazard Ratio (95% CI)	*p* Value
	Harrell’s C-Index (95% CI)		Harrell’s C-Index (95% CI)		Harrell’s C-Index (95% CI)		Harrell’s C-Index (95% CI)	
Age	1.036 (1.002–1.071)	0.024	1.032 (0.984–1.082)	0.169	1.027 (0.967–1.090)	0.361	1.068 (0.977–1.168)	0.118
	0.562 (0.444–0.679)		-		-		-	
Heart rate	1.015 (1.002–1.028)	0.028	1.009 (0.987–1.031)	0.413	1.020 (0.999–1.041)	0.069	1.023 (0.992–1.054)	0.171
	0.670 (0.571–0.768)		-		-		-	
SBP	0.970 (0.953–0.987)	<0.001	0.961 (0.933–0.990)	0.004	0.979 (0.950–1.008)	0.141	0.963 (0.924–1.004)	0.067
	0.687 (0.566–0.807)		0.765 (0.584–0.945)		-		-	
DBP	0.939 (0.912–0.967)	<0.001	0.932 (0.893–0.972)	0.001	0.946 (0.901–0.993)	0.019	0.963 (0.898–1.011)	0.094
	0.717 (0.603–0.831)		0.717 (0.487–0.946)		0.716 (0.594–0.838)		-	
NYHA-FC	5.121 (2.500–10.492)	<0.001	5.814 (1.720–19.653)	0.004	2.864 (0.495–16.565)	0.239	29.917 (5.727–156.269)	<0.001
	0.697 (0.596–0.798)		0.752 (0.605–0.899)		-		0.856 (0.706–1.000)	
Comorbidities, n	1.153 (0.931–1.426)	0.191	1.623 (1.145–2.300)	0.005	0.948 (0.661–1.361)	0.774	0.779 (0.475–1.277)	0.322
	-		0.709 (0.537–0.881)		-		-	
RDW	1.066 (1.032–1.102)	<0.001	1.077 (1.020–1.137)	0.006	1.137 (1.073–1.203)	<0.001	1.006 (0.929–1.090)	0.872
	0.702 (0.571–0.834)		0.722 (0.505–0.938)		0.789 (0.625–0.953)		-	
eGFR	0.958 (0.943–0.974)	<0.001	0.928 (0.898–0.958)	<0.001	0.981 (0.959–1.004)	0.096	0.966 (0.936–0.997)	0.036
	0.792 (0.693–0.892)		0.900 (0.810–0.991)		-		0.756 (0.552–0.959)	
RGR	1.703 (1.485–1.953)	<0.001	1.673 (1.410–1.984)	<0.001	2.700 (1.235–5.902)	0.030	2.527 (1.206–5.298)	0.046
	0.809 (0.696–0.922)		0.931 (0.877–0.984)		0.758 (0.594–0.923)		0.689 (0.368–1.000)	
NT-proBNP	8.750 (3.326–23.018)	<0.001	33.739 (4.632–245.753)	<0.001	2.477 (0.724–8.472)	0.096	108.181 (11.561–1012.243)	<0.001
	0.791 (0.725–0.858)		0.804 (0.700–0.909)		-		0.954 (0.900–1.000)	
	0.809 (0.696–0.922)		0.931 (0.877–0.984)		0.758 (0.594–0.923)		0.689 (0.368–1.000)	

CI, confidence interval; DBP, diastolic blood pressure; eGFR, estimated glomerular filtration rate; HFmrEF, heart failure with mildly reduced ejection fraction; HFpEF, heart failure with preserved ejection fraction; HFrEF, heart failure with reduced ejection fraction; n, number; NYHA-FC, New York Heart Association functional class; NT-proBNP, N-terminal prohormone of brain natriuretic peptide; RDW, red cell distribution width; RGR, red cell distribution width-to-estimated glomerular filtration rate ratio; SBP, systolic blood pressure. Harrell’s C-index was presented for predictors that offered good discrimination ability. *p* value is expressed as per univariate predictor and not the overall model fit computed by the statistical software.

**Table 6:** Cox proportional hazards models (stepwise method) for in-hospital mortality.

Parameter	Entire Cohort n = 627		HFrEF n = 275		HFmrEF n = 170		HFpEF n = 182	
	Hazard Ratio (95% CI)	*p* Value	Hazard Ratio (95% CI)	*p* Value	Hazard Ratio (95% CI)	*p* Value	Hazard Ratio (95% CI)	*p* Value
Age	1.044 (1.010–1.079)	0.010	-	-	-	-	-	-
Heart rate	-	-	-	-	-	-	-	-
SBP	-	-	-	-	-	-	-	-
DBP	-	-	-	-	-	-	-	-
NYHA-FC	3.050 (1.445–6.438)	0.003	-	-	-	-	17.521 (2.404–127.660)	0.004
Comorbidities, n	-	-	1.946 (1.300–2.912)	0.001	-	-	-	-
RDW	-	-	-	-	1.137 (1.073–1.203)	<0.001	-	-
eGFR	-	-	-	-	-	-	-	-
RGR	1.661 (1.391–1.984)	<0.001	1.958 (1.502–2.552)	<0.001	-	-	-	-
NT-proBNP	4.200 (1.533–11.503)	0.005	28.510 (3.641–223.201)	0.001	-	-	342.596 (12.203–9618.118)	<0.001
Model’s overall model fit	<0.001		<0.001		<0.001		<0.001	
Model’s Harrell’s C-index	0.847 (0.768–0.927)		0.963 (0.942–0.985)		0.789 (0.625–0.953)		0.979 (0.966–0.992)	

CI, confidence interval; DBP, diastolic blood pressure; eGFR, estimated glomerular filtration rate; HFmrEF, heart failure with mildly reduced ejection fraction; HFpEF, heart failure with preserved ejection fraction; HFrEF, heart failure with reduced ejection fraction; n, number of cases; NYHA-FC, New York Heart Association functional class; NT-proBNP, N-terminal prohormone of brain natriuretic peptide; RDW, red cell distribution width; RGR, red cell distribution width-to-eGFR ratio; SBP, systolic blood pressure.

**Table 7:** Predictors of 6-month all-cause mortality.

Parameter	Dependent Expected Value (6-Month All-Cause Mortality)		
Entire Cohort (n = 595)	AUC (95% CI)	*p* Value	Cut-Off Point
RDW	0.619 (0.579–0.658)	0.002	>47.4
eGFR	0.715 (0.677–0.751)	<0.001	≤69.84
NT-proBNP (pg/mL)	0.649 (0.610–0.688)	0.001	>3.34
RGR	0.713 (0.675–0.749)	<0.001	>0.69
**HFrEF (n = 262)**			
RDW	0.639 (0.578–0.697)	0.006	>49.5
eGFR	0.671 (0.610–0.727)	<0.001	≤75.29
NT-proBNP (pg/mL)	0.631 (0.570–0.690)	0.001	>3.64
RGR	0.694 (0.634–0.749)	<0.001	>0.69
**HFmrEF (n = 159)**			
RDW	0.660 (0.581–0.733)	0.040	>48.1
eGFR	0.767 (0.693–0.830)	<0.001	≤55.19
NT-proBNP (pg/mL)	0.537 (0.457–0.617)	0.651	>4.01
RGR	0.777 (0.704–0.839)	<0.001	>0.66
**HFpEF (n = 174)**			
RDW	0.501 (0.424–0.577)	0.994	>47.9
eGFR	0.747 (0.675–0.809)	<0.001	≤58.45
NT-proBNP (pg/mL)	0.683 (0.608–0.751)	0.008	>3.32
RGR	0.678 (0.603–0.747)	0.031	>0.97

AUC, area under the curve; CI, confidence interval; eGFR, estimated glomerular filtration rate; HFmrEF, heart failure with mildly reduced ejection fraction; HFpEF, heart failure with preserved ejection fraction; HFrEF, heart failure with reduced ejection fraction; n, number of cases; NT-proBNP, N-terminal prohormone of brain natriuretic peptide; RDW, red cell distribution width; RGR, red cell distribution width-to-estimated glomerular filtration rate ratio.

## Data Availability

Data supporting the findings of this study are available from the corresponding author in accordance with local and national regulations.

## Abbreviations

The abbreviations used throughout this manuscript are as follows:

AF	Atrial fibrillation
AUC	Area under curve
BMI	Body mass index
CAD	Coronary artery disease
CHF	Chronic heart failure
CI	Confidence interval
CKD	Chronic kidney disease
COPD	Chronic obstructive pulmonary disease
DBP	Diastolic blood pressure
eGFR	Estimated glomerular filtration rate
ELOS	Extended length of hospital stay
ESC	European Society of Cardiology
HF	Heart failure
HFA	Heart Failure Association
HFmrEF	Heart failure with mildly reduced ejection fraction
HFpEF	Heart failure with preserved ejection fraction
HFrEF	Heart failure with reduced ejection fraction
HR	Heart rate
IQR	Interquartile range
LOS	Length of hospital stay
LVEF	Left ventricle ejection fraction
NT-proBNP	N-terminal prohormone of brain natriuretic peptide
NYHA-FC	New York Heart Association functional class
RDW	Red cell distribution width
RDW-SD	Red cell distribution width standard deviation
RGR	Red cell distribution width-to-estimated glomerular filtration rate ratio
ROC	Receiver operating characteristic
SBP	Systolic blood pressure
T2DM	Type 2 diabetes mellitus
USA	United States
